# Is the Reason of Increased D-Dimer Levels in COVID-19 Because of ACE-2-Induced Apoptosis in Endothelium?

**DOI:** 10.1177/1076029620935526

**Published:** 2020-07-13

**Authors:** Nil Guler, Fakiha Siddiqui, Jawed Fareed

**Affiliations:** 1 Department of Hematology, Medical School, Pamukkale University, Denizli, Turkey; 2 Department of Pathology, Cardiovascular Research Institute, Loyola University Chicago, Health Sciences Division, Maywood, IL, USA; 3 Department of Pathology and Laboratory Medicine, Department of Pharmacology and Neuroscience, Cardiovascular Research Institute, Loyola University Chicago, Health Sciences Division, Maywood, IL, USA

## To the Editor

Coronavirus disease 2019 (COVID-19) which causes severe acute respiratory syndrome (SARS) was named as SARS-CoV-2 to distinguish it from the previous SARS-CoV.^1^ The rate of acute respiratory distress syndrome (ARDS) is 19.6% in patients with COVID-19 pneumonia and has increased to 61.1% during the time of transfer to intensive care unit (ICU).^2^ This ratio is getting higher in non-survivors (81%) and ARDS is becoming a large concern of death in COVID-19.^3^

Other remarkable findings which are linked to the severity of disease and the risk of mortality in COVID-19 is the increased level of D-dimer. Increment in the levels of D-dimer, Fibrin Degradation Product (FDP) and decrement in antithrombin (AT) has been seen in patients with COVID-19. Changes in D-dimer and FDP were prominent in severe disease as compared to the mild group. Thrombin time has been detected as shorter in critical patients compared to controls.^4^ Prothrombin time and D-dimer values at hospital admission have been shown to be higher in patients who need ICU during the disease process compared to non-ICU patients.^5^ Increased levels of D- dimer have been described as risk factors for development of ARDS and especially for death.^6^ Hospital death has been found to be associated with levels greater than 1 µg/mL of D-dimer on admission.^7^ Gao et al described a cut off value for D-dimer as 0.28 ng/L to predict the severity of disease.^8^ Overt-disseminated intravascular coagulation was determined in 71.4% of non-survivors compared to 0.6% of survivors in later stages of COVID-19 pneumonia.^9^ Anticoagulant treatment with unfractionated heparin and low molecular weight heparin was shown to decrease mortality.^10^

Our hypothesis is that probable mechanisms for the increased D-dimer in COVID-19 may be related to virus life cycle. The apoptotic processes target the endothelial cells of the vascular structure resulting in triggered coagulopathy and the ultimate result of increased D-dimer.

Apoptosis is a programmed cell death and has a critical role in the life cycle of viruses. Viruses can’t live without a host cell and after entering the host cell, they use its organelles to produce structures of virus. To keep alive the host cell, it is important for the virus replication phase after finishing this process, to kill a host cell. This gives an advantage to the virus which can leave the host cell and spread to microenvironments. It is probable that during infection, not only the virus interferes with the apoptotic pathway but also the immune system starts to interact with the apoptotic pathways. Killing infected cells during the replication phase is important.^11–13^ However, it can’t always be explained simply because different viruses interfere with apoptotic pathways in different ways. It is our opinion that increased D-dimer may be the result of apoptotic endothelial cell induced coagulopathy. If apoptotic endothelial injury has a substantial role in pathogenesis, then we have to be concerned about the fate of large endothelial surface in the alveolar vascular bed of lungs. Uncontrolled apoptosis of alveolar endothelial cells can destroy oxygenation in different ways such as micro vascular apoptosis induced thrombi in the alveolar vascular bed and vascular fluid leaks to the lung tissue.

Swine acute diarrhea syndrome coronavirus (SADS-CoV) is a coronavirus that causes severe acute diarrhea. Zhang et al investigated the effects of pan-caspase inhibitors on SADS-CoV infected cells to see the role of apoptosis. They observed decreased SADS-CoV-induced apoptosis and reduced virus replication in SADS-CoV infected cells after the pan-caspase inhibitor.^14^ Li et al studied infectious bronchitis virus (IBV) which is another coronavirus that showed that IBV induces apoptosis at late stages of infection.^15^ Some studies investigated the virus induced apoptosis of endothelial cells. Sumikoshi et al demonstrated that human influenza virus can infect and induce apoptosis in the human umbilical vein endothelial cells (HUVECs).^16^ It is an important finding for apoptosis in human vascular endothelial cells, which may lead to other studies for COVID-19. Another interesting study performed by Anfasa et al showed that Zika virus (ZIKV) infected HUVEC demonstrated increased apoptosis, induction in tissue factor concentration and shortened thrombin generation tests.^17^ Increased levels of D-dimer and thrombosis were also clinically demonstrated in patients with ZIKV and Chikungunya.^18^ These all show us that viruses can infect the endothelium and trigger the secondary hemostasis.

Armstrong et al studies infected human lung microvascular endothelium with human influenza virus. These lung cells underwent apoptosis and caused in an increase in vascular permeability. Inhibition of caspases decreased the influenza-induced endothelial leak.^19^ They reported that these apoptotic findings may help to understand the pathogenesis of acute lung injury in viral pneumonias.

Another inspiring study showed that angiotensin-II-induced apoptosis in primary pulmonary endothelial cells uses AT2 receptor. This study found the results on the activation of intrinsic pathway of apoptotic pathway due to an increased release of cytochrome c from mitochondria and activation of caspase 9. Ongoing apoptosis was blocked by an inhibitor of apoptotic protein Bax.^20^ This finding brings us to whether or not to use angiotensin or AT2 receptor blockers which may help to prevent or recover from COVID-19 because SARS-CoV-2 uses ACE2 for entering to the targeted cells.^21^

There is a probability that after entering the cell, SARS-CoV-2 may start the apoptotic processes in the lung microvascular bed endothelium or coronary endothelium or tissue cells which carry ACE2 receptors. We feel that the microvascular pathology can explain this virus’s different clinical pictures due to tissue damage caused from vascular damage. There is also a risk for other tissue cells that carry ACE2 receptor for apoptotic damages and organ failures. There are supportive histological clues in humans on the results of ongoing apoptosis in endothelial cells. Varga et al showed apoptotic changes in endothelial cells, histologically in COVID-19.^22^

We believe that the relationship between COVID-19 and apoptotic pathways contributes to the observed focal pathogenesis in the lungs and is involved in the progression of severity of the observed acute respiratory distress syndrome. Drugs with anti-apoptotic properties may therefore be helpful in the management of these patients and merit further investigations.
